# Tau-Centric Targets and Drugs in Clinical Development for the Treatment of Alzheimer's Disease

**DOI:** 10.1155/2016/3245935

**Published:** 2016-06-26

**Authors:** Francesco Panza, Vincenzo Solfrizzi, Davide Seripa, Bruno P. Imbimbo, Madia Lozupone, Andrea Santamato, Chiara Zecca, Maria Rosaria Barulli, Antonello Bellomo, Alberto Pilotto, Antonio Daniele, Antonio Greco, Giancarlo Logroscino

**Affiliations:** ^1^Neurodegenerative Disease Unit, Department of Basic Medicine, Neuroscience, and Sense Organs, University of Bari Aldo Moro, Bari, Italy; ^2^Department of Clinical Research in Neurology, University of Bari Aldo Moro, “Pia Fondazione Cardinale G. Panico”, Tricase, Lecce, Italy; ^3^Geriatric Unit & Laboratory of Gerontology and Geriatrics, Department of Medical Sciences, IRCCS “Casa Sollievo della Sofferenza”, San Giovanni Rotondo, Foggia, Italy; ^4^Geriatric Medicine-Memory Unit and Rare Disease Centre, University of Bari Aldo Moro, Bari, Italy; ^5^Research & Development Department, Chiesi Farmaceutici, Parma, Italy; ^6^Psychiatric Unit, Department of Basic Medicine, Neuroscience, and Sense Organs, University of Bari Aldo Moro, Bari, Italy; ^7^Physical Medicine and Rehabilitation Section, “OORR” Hospital, University of Foggia, Foggia, Italy; ^8^Department of Orthogeriatrics, Rehabilitation and Stabilization, Frailty Area, E.O. Galliera NR-HS Hospital, Genova, Italy; ^9^Institute of Neurology, Catholic University of Sacred Heart, Rome, Italy

## Abstract

The failure of several Phase II/III clinical trials in Alzheimer's disease (AD) with drugs targeting *β*-amyloid accumulation in the brain fuelled an increasing interest in alternative treatments against tau pathology, including approaches targeting tau phosphatases/kinases, active and passive immunization, and anti-tau aggregation. The most advanced tau aggregation inhibitor (TAI) is methylthioninium (MT), a drug existing in equilibrium between a reduced (leuco-methylthioninium) and oxidized form (MT^+^). MT chloride (methylene blue) was investigated in a 24-week Phase II clinical trial in 321 patients with mild to moderate AD that failed to show significant positive effects in mild AD patients, although long-term observations (50 weeks) and biomarker studies suggested possible benefit. The dose of 138 mg/day showed potential benefits on cognitive performance of moderately affected AD patients and cerebral blood flow in mildly affected patients. Further clinical evidence will come from the large ongoing Phase III trials for the treatment of AD and the behavioral variant of frontotemporal dementia on a new form of this TAI, more bioavailable and less toxic at higher doses, called TRx0237. More recently, inhibitors of tau acetylation are being actively pursued based on impressive results in animal studies obtained by salsalate, a clinically used derivative of salicylic acid.

## 1. Introduction

The 2015 figures suggested that Alzheimer's disease (AD) may affect over 5.3 million people in the USA [1]. By 2050, the number of new cases of AD per year is expected to grow, resulting in nearly 1 million new cases per year, and the estimated prevalence is expected to range from 11 million to 16 million [1]. In the last three decades, notwithstanding considerable advances in the AD neurobiology and medicinal chemistry, no disease-modifying treatments have been introduced for this devastating and progressive neurodegenerative disease [2]. The neuropathological hallmarks of AD are intracellular neurofibrillary tangles (NFTs) composed of paired helical filaments (PHFs) and straight filaments (SFs) mainly constituted of hyperphosphorylated tau protein, a microtubule associated protein (MAP), neuropil threads (NTs), dystrophic* neurites*, and extracellular deposits of *β*-amyloid (A*β*) as the major component of senile plaques (SPs) in the brain. These neuropathological hallmarks of AD strongly influenced recent therapeutic approaches, with a large portion of the many therapeutic approaches under development for AD treatment directed against the production and accumulation of A*β* [3]. However, several drugs targeting A*β* with different mechanisms of action have failed to demonstrate efficacy in randomized clinical trials or their development has been halted [4, 5]. For the amyloid-based approach, passive anti-A*β* immunization is the most advanced strategy for treating AD, and solanezumab, a monoclonal antibody directed at the mid-region of A*β*, also failed but suggested some beneficial cognitive effects in mildly affected patients [4]. A Phase III study with a planned size of 2100 mild AD patients is ongoing to confirm these potential benefits. Solanezumab is also being tested in a prevention study in asymptomatic older subjects, who have positive positron emission tomography (PET) scans for brain amyloid deposits [6]. Two other monoclonal antibodies, gantenerumab, which preferentially binds to fibrillar A*β*, and crenezumab, which preferentially binds to soluble, oligomeric and fibrillar A*β* deposits, are being tested in secondary prevention trials in presymptomatic subjects with autosomal dominant AD mutations [4, 6]. These ongoing secondary prevention trials will tell us if A*β* really plays a crucial role in the pathophysiology of AD. In fact, notwithstanding the preeminence assigned to A*β*, one crucial point was that large numbers of amyloid SPs can occur in the course of normal ageing without any evidence of clinical dementia. Given the repeated failures of trials targeting the A*β* pathway in mild or moderate AD [4], there is increasing interest in the possibility that tau-targeted compounds could have therapeutic utility in AD, particularly tau aggregation inhibitors (TAIs) [5, 7, 8]. The aim of this paper was to provide a comprehensive review of tau-directed drugs for the treatment of AD, with a particular focus on TAIs and the most clinically advanced of these compounds, that is, leucomethylthioninium (LMT, leucomethylene blue (MB), LMTX*™*, TRx0237, TauRx Therapeutics Ltd., Republic of Singapore), a second-generation TAI for the AD treatment. TRx0237 shares the same active ingredient and mode of action of another first-generation TAI, that is, methylthioninium (MT, Rember*™*, TRx-0014, TauRx Therapeutics Ltd., Republic of Singapore), of which is the reduced form, designed to have improved bioavailability and tolerability. The chloride salt of oxidized MT^+^ is methylthioninium chloride (MTC or MB).

## 2. Pathophysiology of Tau Protein in Alzheimer's Disease

Among pathological hallmarks of AD, the intracellular NFTs contain two aggregated tau species, hyperphosphorylated PHFs of MAP tau (or tau) and SFs. Tau is a 50–75 kDa protein with six different splice variants, referred to as 0N3R, 1N3R, 2N3R, 0N4R, 1N4R, and 2N4R [9, 10]. A short segment of tau protein, referred to as the PHF core, from the repeat region of the molecule is an integral structural constituent of the PHF [11]. Abnormal phosphorylation/hyperphosphorylation occurs in tau protein in AD, beginning to pair up with other threads of tau into PHFs and tangle together, resulting in the movement of tau proteins from axons to the somatodendritic compartment of neurons, causing disintegration of microtubules, collapse of neuron's transport system, and formation of extremely insoluble aggregates. These changes are presumed to disrupt neuronal communication and lead to cell death [12]. NFTs are generated intracellularly, but when neurons die, the only NFTs remaining are “ghost tangles” which are localized outside of cells where the host neuron has died. Ghost tangles are a common finding in AD patients which can occur also in preclinical stages [13]. In this preclinical phase of AD, the earliest involved neurons are those in the locus coeruleus, and the subcortical tau lesions then reach the noradrenergic coeruleus neurons of the contralateral brainstem, so that the pathological process becomes symmetrical soon after its onset. Thereafter, additional nuclei with diffuse cortical projections become involved [14]. H. Braak and E. Braak demonstrated that appearance of tau pathology in AD occurs in a characteristic pattern of development in six stages, with NFTs and NTs appearing first in the entorhinal cortex (stages I and II), followed by hippocampal (stages III and IV), and neocortical areas (stages V and VI) [13–15]. A corresponding staging for A*β* deposition was compared with tau staging, with three levels of increasing A*β* deposits (stages A–C), in a large autopsy case series of subjects between the ages of 25 and 95 years [16]. These findings suggested that tau aggregation precedes A*β* deposits by about three decades [16], confirming earlier reports showing the same pattern [17, 18].

The exact mechanisms by which tau protein becomes a nonfunctional entity are under debate. Tau pathology in AD is principally characterized by abnormal phosphorylation/hyperphosphorylation of tau proteins, but also proteolytic cleavage (truncation), glycosylation, nitration, acetylation, O-GlcNAcylation, ubiquitination, and other abnormal posttranslational modifications are responsible for altered tau structure in this devastating neurodegenerative disease [11, 19–25]. All these molecular events are associated with the formation of PHFs and the appearance of NFTs. In particular, abnormal phosphorylation/hyperphosphorylation, acetylation, and truncation are further supported as pathological events by* in vitro* experiments [22, 26–29], demonstrating that these modifications increase fibrillization of tau and induce cell toxicity. Truncation/proteolytic cleavage of tau protein, as an alternative mechanism involving in the abnormal aggregation of tau, was proposed after extensive biochemical analysis of the PHF core [11, 21], with prion-like properties* in vitro*. Until today, identification of the enzyme that produces this abnormal posttranslational modification is uncertain. Caspases, which are apparently elevated in AD brain [30, 31], are likely involved in the proteolytic processing of tau protein. The repeat domain of tau is able to catalyse and propagate the conversion of normal soluble tau into accumulations of the aggregated and truncated oligomeric forms [5]. In fact, hyperphosphorylated tau proteins bind together and form oligomeric tau, from dimers to octamers [32]. Both hyperphosphorylated tau by itself and oligomeric tau are involved in synaptic loss, as observed in the wild-type human tau transgenic mouse [25, 33]. Indeed, protein aggregates may in general be protective in neurodegeneration by sequestering dispersed small aggregates, oligomers, or misfolded proteins, minimizing their toxicity and eventually facilitating their clearance by proteasomal activity or autophagy [34–36], a model that remains to be validated with respect to tau protein and AD [37]. However, proteolytically stable tau oligomers are able to propagate between neurons and initiate the cascade of self-propagating misfolded proteins from neuron to neuron [38, 39]. Therefore, the tau pathology of AD can be understood as a self-propagating ‘‘prionosis,” reflecting degrees of spread of tau that may form an endopathogenic species transmitting neurodegeneration from one cell to the next throughout the brain [40]. On this basis, vaccination of mice in experimental models of tauopathy and synucleinopathy, involving intracellular proteins, has showed promising findings [41, 42].

## 3. Tau-Based Drugs for the Treatment of Alzheimer's Disease

In AD, given the confirmed link existing between NFT topography and clinical phenotype [43], and the abnormal posttranslational modifications of tau protein linked to the disease [11, 19–25], therapies targeting NFTs and tau protein may have potential application as drug targets against neurodegeneration [44–46], although their development has lagged behind drugs targeting A*β*. At present, therapies targeting tau aim to reduce, stabilize, or prevent aggregation or hyperphosphorylation of the protein [44–46]. In particular, several therapeutic approaches with a disease-modifying potential have been suggested: (1) inhibition of tau phosphorylation (with the inhibition of tau kinases or the activation of tau phosphatases); (2) increase of microtubule stabilization; (3) increase of tau clearance and (4) inhibition of tau aggregation. Some of these approaches have actually reached the clinic [7].

Abnormal phosphorylation of tau protein may play a critical role in the pathogenesis of NFT degeneration, with the balance between kinases and phosphatases disturbed in AD, leading tau protein to become detached from microtubules, secondarily to aggregate. There is approximately a four- to fivefold higher level of total tau in AD brain compared to that of age-matched healthy brains and this increase is all in the form of the abnormally hyperphosphorylated tau [47]. In AD, the abnormal phosphorylation of tau could be, but not mutually exclusive, the result of upregulation of tau kinases or downregulation of tau phosphatases [9]. In this scenario, a tau-based therapeutic approach would target a kinase particularly responsible for a pattern of phosphorylation causing reduced microtubule stability.

Tau protein kinases are grouped into three main classes: proline-directed protein kinases (PDPK), protein kinases non-PDPK, and tyrosine protein kinases (TPK) [48]. Among these enzymes, the kinases with the most important role in phosphorylation of tau protein in the brain include glycogen synthase kinase 3*β* (GSK-3*β*), cyclin-dependent kinase 5 (CDK5), cAMP-dependent protein kinase (PKA), and calcium/calmodulin-dependent kinase II (CaMKII) [49]. GSK-3*β* may play a major role in regulating tau phosphorylation in both physiological and pathological conditions. Interactions between GSK-3*β* and CDK5 also exist and will require further evaluation to optimize treatments aimed at these kinases [50, 51]. Despite the challenges faced by this approach with respect to toxicity and specificity, a number of efforts are underway to develop kinase inhibitors. In particular,* in addition to* a series of compounds directed at kinases of the PDPK and non-PDPK groups in preclinical development that should be tested in* in vivo* studies [48, 52], one GSK-3*β* inhibitor, tideglusib (NP031112, NP-12, Nypta®, Zentylor*™*, Noscira SA, Madrid, Spain), a drug which belongs to the thiadiazolidinone family, was in clinical trials for AD and progressive supranuclear palsy (PSP) [5, 7, 53]. In a previous Phase IIa trial, tideglusib was orally administered at escalated doses of 400 up to 1000 mg/day for 20 weeks to 30 patients with mild to moderate AD and the active group showed positive trends in four out of five clinical scales and had significantly better response on the Mini-Mental State Examination (MMSE), with asymptomatic elevation of transaminases, reversed with withdrawal of the drug [54]. The ARGO study, a subsequent six-month, Phase IIb trial, was conducted to assess safety and efficacy of tideglusib in mild to moderate AD patients with the 15-item modified version of the Alzheimer's Disease Assessment Scale (ADAS-cog_15_) as the principal outcome measure. However, the results demonstrated no statistically significant findings, although the drug was well tolerated with diarrhea and asymptomatic transaminase elevations as the only side effects [55]. There are no current FDA approved trials ongoing for treating AD with tideglusib. Activation of phosphatases, in particular protein phosphatase 2A (PP2A), has also been proposed as a possible alternative strategy to kinase inhibition for reducing tau phosphorylation [44, 49, 56]. Multiple PP2As exist and inhibition of these phosphatases results in hyperphosphorylation of tau, formation of NFT-like structures, and memory impairment in animal models [57–59]. Drugs increasing the activity of PP2As, probably through the endogenous proteins that inhibit their activity, have the therapeutic potential for treating AD [60, 61], but no clinical trials with PP2A activators have been started yet.

Among tau-based anti-AD drugs, several microtubule-stabilizing agents have been tested and the studies carried out have provided proof of concept that compounds with the ability to stabilize microtubules may have therapeutic potential for the treatment of AD and other neurodegenerative diseases [62], given that tau detachment from microtubules results in loss of its normal microtubule-stabilizing function, probably leading to axonal transport impairment and eventually to synaptic dysfunction. Some antimitotic compounds such as paclitaxel (Taxol, Bristol-Myers Squibb Company, New York City, USA), epothilone D (Epo D, BMS-241027, Bristol-Myers Squibb Company, New York City, USA), or TPI-287 (Cortice Biosciences, New York City, USA, formerly Archer Biosciences) have been used in tau transgenic animals for their microtubule-stabilizing activity [7, 63, 64], but at present, these compounds did not reach the clinic* due to* toxic side effects (paclitaxel) or have been discontinued for AD (epothilone D) or are in Phase I of clinical development (TPI-287) for mild to moderate AD [65] (Table 1) and primary four-repeat tauopathy, corticobasal degeneration (CBD), CBD syndrome, and PSP [66]. In particular, in a preventative study, epothilone D was administered weekly for 3 months to young PS19 tau Tg mice that initially lacked significant tau pathology, preventing the axonal microtubule loss and dystrophy, as well as spatial learning deficits, that manifested as these mice developed forebrain tau pathology with age [67]. In another preclinical study, in both young and old animals of the PS19 tauopathy model, in which tau pathology is developing or well established, respectively, epothilone D reversed behavioral and cognitive deficits, cleared tau pathology, and increased hippocampal neuronal integrity [63]. Based on these encouraging findings, in February 2012, Bristol-Myers Squibb started a Phase I trial to evaluate the tolerability and pharmacology of epothilone D in 40 patients with mild AD, comparing 0.003, 0.01, and 0.03 mg/kg infused once a week for nine weeks to placebo [68]. The study ended in October 2013, but evaluation of epothilone D for AD was subsequently discontinued.

Among microtubule-stabilizing agents, davunetide (NAPVSIPQ, NAP, AL-108, Allon Therapeutics Inc., Vancouver, Canada, Paladin Labs Inc., Montreal, Canada), an eight-amino acid peptide (with NAP representing the first three amino acids in the peptide) derived from the activity-dependent neuroprotective protein (ADNP), has demonstrated the potential to decrease tau phosphorylation and A*β* levels in animal models [69]. In particular, NAP stabilizes microtubules and reduces hyperphosphorylated tau levels [70] and in a mouse model of amyotrophic lateral sclerosis (ALS) it protected against impairments in axonal transport [71], suggesting that reduction of tau hyperphosphorylation, stabilization of microtubules, and neuroprotective effects may be beneficial to prevent disease progression. An intranasal formulation of davunetide was tested in Phase II clinical trials for both mild cognitive impairment (MCI) and PSP, given that intranasally administered NAP treatment can cross the blood-brain barrier (BBB). In 2007-2008, the Phase II trial in 144 subjects with MCI demonstrated a statistically significant improvement in memory performance compared with placebo at eight weeks and 16 weeks, but not 12 weeks, with well-tolerable side effects [72]. However, the results of the Phase II/III trial in the pure tauopathy PSP were unimpressive [73], suggesting intervention at early stages of the disease [62]. This result halted, for the time being, clinical development of davunetide. This decision also prompted a halt to recruitment into an ongoing safety and biomarker trial, begun in 2010, of davunetide in frontotemporal lobar degeneration (FTLD) with predicted tau pathology, CBD syndrome, or PSP [73]. An intravenous formulation of davunetide also exists (AL-208) and this version of the drug was tested between 2006 and 2008 in a Phase II trial of the safety and efficacy of a single 300 mg IV dose on cognitive impairment following coronary artery bypass surgery [74], with no published results.

Recent efforts to develop safe and efficacious anti-A*β* immunotherapy using A*β* peptide as an immunogen in active vaccination approaches or anti-A*β* antibodies for passive vaccination may be translated to the development of a tau-based immunotherapy [45]. Clearance of extracellular misfolded tau protein may prevent the transmission and spreading of tau pathology throughout the brain. Active immunization of wild-type mice with recombinant unphosphorylated full-length human tau protein led to encephalomyelitis with neurological and behavioral deficits, axonal damage, and inflammation [75], suggesting a neurotoxic potential of tau immunization. However, the feasibility of this approach was later demonstrated with a 30-amino acid tau phosphopeptide spanning amino acids 379–408, including phospho-Ser at positions 396 and 404, in two different transgenic mouse models of disease, the JNPL3 (P301L) and htau/presenilin 1 (PS1) lines [41, 76], which both resulted in a specific antibody response, reduced tau burden, and attenuation in the severity of behavioral and cognitive phenotypes [77]. Among active vaccines in clinical trials, AADvac1 (Axon peptide 108 conjugated to KLH, Axon Neuroscience, Bratislava, Slovak Republic) was the first anti-tau vaccine to enter clinical trials and it is a synthetic peptide derived from amino acids 294 to 305 of the tau sequence, that is, KDNIKHVPGGGS, coupled to keyhole limpet hemocyanin (KLH) through an N-terminal cysteine, and administered with an Alhydrogel alum adjuvant. In transgenic tau rats, the vaccine reduced tau pathology and associated behavioral deficits [78]. AADvac1 was designed to target misfolded tau in AD, and its safety, tolerability, and efficacy have been evaluated in a first-in-man Phase I clinical trial conducted in three centers in Austria on 30 patients with mild to moderate AD, completed on March 2015 [79] (Table 1). Two withdrew due to adverse events, of which one (a viral infection followed by epileptic seizure) was considered to be possibly related to the vaccine. Unfortunately, the double-blind, placebo-controlled portion of the study lasted only 12 weeks and the study evaluated only one dose of the vaccine (40 *μ*g). No data on cerebrospinal fluid (CSF) biomarkers were reported. These deficiencies limit the interpretability of the results both in terms of safety and on target engagement. The subsequent 12-week open label portion of the study is of limited information [80]. Patients completing this 24-week study had the option to enter a further 18-month open label extension (FUNDAMANT) [81] (Table 1). A separate 24-month Phase II study in 185 patients with mild AD and a magnetic resonance imaging (MRI) consistent with this diagnosis was planned to start on March 2016. This study will compare 8 subcutaneous injections of 40 *μ*g of AADvac1 with the adjuvant aluminum hydroxide to placebo. The primary outcome will be safety, and secondary outcomes will include cognitive and clinical batteries as well a measure of immunogenicity. Fluorodeoxyglucose (FDG) PET, MRI volumetry, and CSF biochemistry were set as exploratory outcomes (ClinicalTrials.gov Identifier: NCT02579252, ADAMANT) [82] (Table 1).

The vaccine ACI-35 (AC Immune AG, Lausanne, Switzerland and Janssen Pharmaceuticals, Beerse, Belgium) is a liposomal-based 16-amino acid tetrapalmitoylated phospho-tau peptide with specific amino acid areas incorporated into the vaccine including phosphorylated S396 and S404 residues that also provides active immunization. It elicits a rapid immune response against the immunogen in wild type and transgenic JNPL3 (P301L) mice, resulting in a mild reduction of hyperphosphorylated pathological tau and tau pathology by immunohistochemical characterization and increased IgG titers and motor function of vaccinated mice [83]. ACI-35 also demonstrated a good safety profile for human studies, with no adverse inflammatory response [83]. Currently, a Phase Ib trial is underway in mild to moderate AD to assess safety profile along with secondary outcomes including biomarkers, functional, and clinical change (Table 1), but details are not available and this trial is not listed in ClinicalTrials.gov or the World Health Organization's clinical trial registry.

For passive vaccination, anti-tau oligomer antibodies may be ideal candidates for treating AD [84], similar to the ones developed for A*β* [85], with exciting opportunities to validate anti-tau oligomer immunotherapeutic approaches in animal models. In the first program to demonstrate the efficacy of tau-based immunotherapy, this approach has been tested by injecting anti-phospho-tau antibody PHF1, which recognizes the pS396/pS404 epitope, intraperitoneally to JNPL3 (P301L) tau transgenic mice, with preliminary findings indicating that treated animals showed decreased tau pathology and functional impairment [86]. Similar effects were obtained also with other antibodies against the pS396/pS404 epitope [87, 88]. Several tau antibodies are currently in early clinical development as therapies for AD and other tauopathies [45]. Among these antibodies, RG7345 (RO6926496, MAb86, F. Hoffmann-La Roche Ltd., Basel, Switzerland) is a human monoclonal antibody targeting a specific tau phosphorylated epitope at site pS422, which is prominent in neuronal dendrites [89, 90] and linked to the relocalization of tau protein away from microtubules and toward the somatodendritic compartment of the neuron [89]. Furthermore, in a triple transgenic mouse model of AD, the passive administration of the antibody demonstrated a reduced accumulation of tau pathology with intracellular clearance of tau antibody complexes [90]. In January 2015, a Phase I, single-ascending-dose study in 48 healthy young men in the United Kingdom started, comparing the safety and pharmacokinetic measures of six different doses to placebo, all infused intravenously [91] (Table 1). Finally, BMS-986168 (IPN007, Bristol-Myers Squibb Company, New York City, USA), although not currently approved for AD but only for PSP [92], is a humanized monoclonal antibody directed toward extracellular, N-terminally fragmented forms of tau (eTau), which were originally isolated from familial AD patient-derived pluripotent stem cells. A recent study demonstrated a correlation between eTau and A*β* both* in vitro* and in two transgenic* in vivo* mice models, with a reduction in A*β* that occurs when eTau is targeted with an antibody [93]. Secreted forms of tau were reported to cause neuronal hyperactivity, which could, in turn, increase A*β* production, fueling a feed-forward cycle [93]. In December 2014, a Phase I, single-center, single-ascending-dose study in 48 healthy volunteers in Texas started. This first human trial will assess safety parameters for up to eight months after administration of a single infusion of BMS-986168 [94].

## 4. Covalent and Noncovalent Tau Aggregation Inhibitors for the Treatment of Alzheimer's Disease

Among several tau-directed approaches in AD, small molecular weight compounds developed to inhibit formation of tau oligomers and fibrils by blocking tau-tau aggregation have already been tested in humans [5, 7, 95, 96]. In cell-based and/or* in vitro* screening assays, several classes of agents that may act to prevent tau aggregation have been identified, including but not limited to polyphenols [80], porphyrins [80], phenothiazines [97], benzothiazoles/cyanines [98, 99], N-phenylamines [100], thioxothiazolidinones (rhodanines) [101], phenylthiazole-hydrazides [102], anthraquinones [103], and aminothienopyridazines (ATPZs) [104]. However, for many TAIs there is a lack of evidence of efficacy* in vivo* for inhibiting tau aggregation. Currently, TAIs fall into two broad mechanistic classes, with the first class corresponding to covalent TAIs, that is, agents that either covalently modify tau directly or foster formation of covalent bonds within or between tau proteins to yield aggregation-incompetent products [95]. Covalent TAIs can attack any or all species in an aggregation pathway but appear to be especially efficacious modifiers of tau monomers [95]. Among covalent TAIs, oleocanthal, a natural product aldehyde, reacts with epsilon amino groups of lysine residues [105, 106], including residues residing in the microtubule binding repeat region, to form imines. In addition, other natural polyphenols are covalent TAIs, such as oleuropein aglycone [107], abundant in the extra virgin olive oil, or green tea-derived (−)-epigallocatechin gallate (EGCG) [108]. Other redox-active compounds, including the nonneuroleptic phenothiazine MB, that is, MTC, can also modulate cysteine oxidation when incubated in the absence of exogenous reducing agents [109]. High concentrations of reduced sulfhydryl groups in the form of glutathione normally maintain a reducing intracellular environment [110], and therefore compounds acting solely through this mechanism could have low potency and efficacy* in vivo*. In general, covalent mechanisms of tau aggregation inhibition in AD are predicted to have low utility* in vivo* [111]. However, dimethylfumarate, an electrophile capable of reacting covalently with cysteine sulfhydryls, was approved for oral treatment of multiple sclerosis [112], suggesting that electrophilic compounds acting through covalent inhibitory mechanisms can be useful therapeutic agents.

The second broad class of TAIs interacts with tau species noncovalently, through multiple mechanisms, and with different structures [95, 113]. Among different mechanisms, small molecules can interact directly but transiently with natively unfolded tau protein monomer [95]. For example, curcumin has been reported to increase the reconfiguration rate (i.e., a rapid rate of interconversion between aggregation competent and incompetent conformations) of *α*-synuclein, such that occupancy of assembly competent conformations is minimized [114]. Because tau aggregation is sensitive to curcumin conjugates [115], this mechanism may be relevant also for tau protein. Noncovalent TAIs also may act by blocking formation of steric zipper structures common to cross-*β*-sheet forming peptides. Short segments of amyloidogenic sequences have been crystallized in forms that exhibit similar properties as their full-length counterparts [116]. Furthermore, tau filament formation can be inhibited by sequestering tau in the form of stable off-aggregation pathway oligomers. For example, phthalocyanine tetrasulfonate, a cyclic tetrapyrrole, interacts directly with tau monomers to form SDS-stable oligomers [117]. Similarly, in a study of *α*-synuclein aggregation, polyphenol, phenothiazine, polyene macrolide, porphyrin, and Congo red derivatives were found to stabilize SDS- and Sarkosyl-insoluble oligomers [118]. SDS-stable oligomers composed of full-length tau also rapidly form at low micromolar concentrations in the presence of cyanine, triarylmethine, rhodanine, and phenothiazine TAIs [80, 111]. Since tau can coaggregate with other proteins, including microtubule associated proteins and alpha-synuclein [119], TAIs may work through binding to these proteins. Indeed, numerous polyphenols have been identified that inhibit aggregation of a wide variety of amyloidogenic peptides including tau and *α*-synuclein [113], but no studies with selective TAIs are currently available to support this hypothesis.

## 5. Tau Aggregation Inhibitors in Clinical Development for the Treatment of Alzheimer's Disease: Preclinical Studies of Methylthioninium and Derivatives

TAIs are divided into covalent and noncovalent molecules depending on their way to interact with tau protein. The chemical structure of noncovalent TAIs differs significantly in terms of scaffold [95]. Structure-activity relationships (SARs) were established within specific chemical series [120, 121]. Like common dyes, most TAIs absorb electromagnetic radiation in the visible spectrum, a property linked to the property of delocalizing *π*-electron distribution [122]. Ligand polarizability correlates with tau aggregation inhibitory potency within specific chemical series of cyanine, phenothiazine, arylmethine, and rhodanine derivatives [111]. MB or MTC (Rember) is and old dye repurposed as medical treatment of tau pathologies [123]. Chemically, MTC is a tricyclic phenothiazine derivative [124] and exists in equilibrium between reduced (LMT) and oxidized form (MT^+^). Under physiological conditions, it is present as a cation (MT^+^) and formulated as a chloride salt (commonly known as MB). MTC may be reduced by nicotinamide adenine dinucleotide phosphate (NADPH) or thioredoxin to give LMT (leuco-MB), an uncharged colorless compound (methylene white). MTC is excreted in the urine as a mixture of MTC, LMT, and demethylated metabolites, for example, azure B and azure A [125]. MTC has been used to treat malaria [126], methemoglobinemia [123], and depression [127]. MTC efficiently crosses the BBB [128] and selectively penetrates neurons after systemic administration, particularly hippocampal cells [129]. At present, MTC and its derivatives represent the most advanced TAIs in clinical development for the treatment of AD. MTC has been shown to interfere with the tau-tau binding necessary for aggregation [97]. In a cell-based model of inducible tau aggregation, the inhibitory constant of MTC was found to be 123 nM [5]. Other studies reported quite different* in vitro* inhibitory potency (IC_50_) varying from 1.9 *μ*M [80] to 3.5 *μ*M [99]. The estimated trough brain concentration of MT (Rember) and its active metabolites in the human brain at the 138 mg/day dose was 0.18 *μ*M [130]. This value appears to be in the range of the* in vitro* IC_50_ values for dissolution of PHFs (0.16 *μ*M) and the calculated intracellular Ki for TAI activity (0.12 *μ*M) [131] but not in the range of IC_50_s of other* in vitro* [80] and cell-based [99] studies. In tau transgenic mouse models, MT levels in the brain followed a sigmoidal concentration-response relationship over a 10-fold range (0.13–1.38 *μ*M) after oral administration of 5–75 mg/kg for 3–8 weeks [132]. Alternative mechanisms of action have been proposed for MT [5] including inhibition of microtubule assembly [104] that requires IC_50_ of 50 *μ*M [5, 104]. However, the dose required to achieve inhibition of microtubule assembly with MTC would be about 50 g of MTC/day [5], exceeding the median lethal dose (LD_50_) for MTC in several species. Similarly, it has been proposed that MTC may reduce endogenous production of tau protein [133], but EC_50_ for this effect is 10 *μ*M, requiring a human clinical dose of 9 g of MTC/day, a dose that could not safely be administered in humans. It has been also proposed that MTC could affect tau phosphorylation via inhibition of Hsp70 ATPase [134], but again EC_50_ for this effect is 83 *μ*M, with a theoretical dose in humans of 75 g MTC/day.

Recent* in vivo* and* in vitro* studies have suggested that MTC may reduce tau protein aggregates in AD through proteasomal [135] and macroautophagic [136, 137] degradation of the protein. Other potential effects of MTC are oxidation of cysteine sulfhydryl groups in the tau repeat domain preventing formation of disulphide bridges to keep tau monomeric [138], acetylcholinesterase inhibition [139], nitric oxide synthase inhibition [140], noradrenaline uptake inhibition [141], glutamatergic inhibition [142], monoamine oxidase B inhibition [143], guanylate cyclase inhibition [140], and inhibition of the aggregation of A*β* peptides [80, 97, 144], stimulation of A*β* clearance [145], improvement of brain metabolism [146–150], improvement of astrocyte cellular respiration [151], improvement of brain mitochondrial amyloid-binding alcohol dehydrogenase (ABAD) functions [150], improvement of mitochondrial antioxidant properties [152, 153], improvement of the Nrf2/antioxidant response element (ARE) [154–156], antagonism of *α*7-nicotinic acetylcholine receptors [157], inhibition of *β*-secretase activity [149], enhancement of mitochondrial oxidation [158], and inhibition of monoamine oxidase A [143]. However, the clinical relevance of these potential effects is doubtful. On the other hand, there are only a few reports on the effect of MTC on tau aggregation* in vivo *[135, 136, 159–161]. In one study, MTC did not alter abnormal tau phosphorylation and failed to inhibit tau-dependent neuronal cell toxicity in zebrafish [159]. In another study, MTC treatment reduced detergent-insoluble phosphorylated tau levels in the JNPL3 (P301L) tau transgenic mice [160]. Treatment of 3-month-old rTg4510 mice for 12 weeks with oral MTC prevented behavioral deficits and reduced soluble tau levels in the brain [135]. JNPL3 (P301L) mice treated with MTC for 2 weeks showed reduced soluble tau levels without affecting insoluble tau levels [136]. These studies indicate that MTC treatment may reduce soluble tau levels and prevent cognitive decline when treatment begins at a time point before NFTs are present in the brain [135]. A recent study suggested that 6 weeks of oral treatment with MTC did not reverse established NFT pathology in the rTg4510 mouse model of tauopathy [161]. Some studies reported a generalized antiaggregation effect for MTC against aggregation-prone proteins, such as prion protein [162], *α*-synuclein, and transactivation response (TAR) DNA-binding protein of Mr 43 kDa (TDP-43) [163, 164]. This further activity of MTC has potential relevance for the treatment of ALS and FTLD [165].

## 6. Clinical Efficacy and Safety of Methylthioninium and Derivatives

A double-blind, randomized, placebo-controlled study evaluates the safety and explorative efficacy of MT (Rember) given doses of 69 mg, 138 mg, and 228 mg/day (equivalent to 30 mg, 60 mg, and 100 mg MTC) for 24 weeks to 321 mild to moderate AD patients who were not taking acetylcholinesterase inhibitors (AChEIs) or memantine (ClinicalTrials.gov Identifier: NCT00515333) (Table 1). The primary efficacy outcome of the study was the change in the ADAS-cog at 24 weeks relative to baseline. The effects of treatments on regional cerebral blood flow (rCBF) decline were determined in a subgroup of 135 patients using hexamethyl-propyl-amine-oxime single photon emission computed tomography (HMPAO-SPECT). At the end of the 24-week, double-blind, placebo-controlled treatment period, patients had the option to enter two consecutive open label extensions of 26 and 48 weeks, respectively [166]. At 24 weeks, there were not significant differences between treatment groups compared to placebo in any of the efficacy variables.* Post hoc* subgroup analyses revealed that in moderately affected patients there was significant treatment benefit of the intermediate dose of 138 mg/day compared to placebo on the ADAS-cog scale (5.42 points, *p* = 0.047). In mildly affected patients, there was a significant beneficial effect of the 138 mg/day compared placebo on the all regions other than the left frontal lobe (1.97%, *p* < 0.001) [166].

A total of 111 patients completed the first open label extension of 26 weeks (ClinicalTrials.gov Identifier: NCT00684944) [167] (Table 1). At 50 weeks, the mean change of ADAS-cog score of the 138 mg/day dose group was better than the mean change of patients initially receiving placebo for 24 weeks and then 152 mg/day for 26 weeks (2.8 and 5.2 points in mild and moderate patients, resp.). The most commonly reported adverse events (incidence ≥ 5%) in MTC-treated subjects included gastrointestinal disorders (primarily diarrhea), renal and urinary disorders (primarily dysuria and frequency), and falls [166]. No changes of clinical significance were observed in any routine clinical chemistry parameters in any treatment group. Treatment with MTC produced dose-dependent decreases in red cell count and hemoglobin and increases in methemoglobin. There were 4 cases (of 307 exposed to MTC) with methemoglobin greater than 3.5% (a threshold set for withdrawal of treatment) which resolved on cessation of treatment [166]. The authors of the study reported that the delivery of the highest dose was impaired due to dose-dependent dissolution and absorption factors of the 100 mg MTC gelatin capsule formulation [130]. At present, MTC (Rember) was discontinued for AD treatment.

## 7. Pharmacokinetic, Preclinical, and Clinical Studies with Leucomethylthioninium and Derivatives

To the light of this functional and clinical dissociation identified for MT for AD treatment, TauRx Therapeutics developed the synthesis of a novel chemical entity, TRx0237 (LMTX), a second-generation TAI that is a stabilized, reduced form of MTC, in which LMT is available in an anhydrous crystalline form as the dihydromesylate or the dihydrobromide that is stable in an oxygen atmosphere [131]. X-ray crystal structure determinations of TRx0237 demonstrated that the nitrogen atoms at positions 3 and 7 have tetrahedral geometry [131], distinguishing it from LMT, in which the corresponding nitrogen atoms are in a trigonal pyramidal geometry and not protonated. Synthesis of LMT has to be performed under an inert atmosphere because it rapidly oxidizes on exposure to air, while TRx0237 can be manufactured in bulk without the need for deoxygenation and remains stable for at least 2 years in air atmosphere. Thus, TRx0237 represents a new chemical entity that is distinct from both MTC and LMT, and it is highly soluble and exists as a single polymorph, in contrast to MTC, which is far less soluble and demonstrates heterogeneous polymorphism. TRx0237 remains stable for at least 2 years in air atmosphere, is highly soluble, and exists as a single polymorph [168]. An* in vitro *study showed the ability of TRx 0237 in disrupting PHFs isolated from AD brain tissues at the concentration at 0.16 *μ*M [131]. The comparative* in vivo* pharmacological efficacy of MTC and LMT salts (TRx0237: 5–75 mg/kg with oral administration for 3–8 weeks) was assessed in these two novel transgenic tau mouse lines modeling cognitive and motor endophenotypes of AD and FTLD tauopathies [169], namely, impairment in spatial learning (L1) and motor learning (L66), respectively [132]. In this* in vivo* study, both MTC and TRx0237 dose-dependently rescued the learning impairment and restored behavioral flexibility in a spatial problem-solving water maze task in L1 (minimum effective dose: 35 mg MT/kg for MTC, 9 mg MT/kg for TRx0237) and corrected motor learning in L66 (effective doses: 4 mg MT/kg) [132]. Both compounds reduced the number of tau-reactive neurons, particularly in the hippocampus and entorhinal cortex in L1 and in a more widespread manner in L66. The relative superiority of TRx0237 compared with MTC appears to be therefore more likely due to factors related to absorption, metabolism, and distribution, rather than to inherent pharmacodynamic differences.

No direct information on Phase I trials is available. A 4-week Phase II safety study of 250 mg/day of TRx0237 in 9 patients with mild to moderate AD already taking AChEIs and/or memantine began in September 2012 but was terminated in April 2013, reportedly for administrative reasons (ClinicalTrials.gov Identifier: NCT01626391) [170] (Table 1). Currently, three Phase III trials with TRx0237 are ongoing plus an open label extension study (Table 1). The first study compares a single 200 mg/day dose of the compound to placebo in 700 patients with a diagnosis of either all-cause dementia or AD mild enough to score above an MMSE of 20 (ClinicalTrials.gov Identifier: NCT01689233) [171] (Table 1). Started in November 2012, this trial is ramping up to involve more than 90 sites in North America and Europe, using as primary outcome measures of efficacy the ADAS-Cog 11 and the ADCS-CGIC scales. Temporal lobe brain metabolism is measured by 18F-fluorodeoxyglucose- (FDG-) PET imaging and safety parameters. The second Phase III trial compares 150 and 250 mg/day of TRx0237 to placebo in 833 patients with mild to moderate AD with an MMSE of 14 or higher (ClinicalTrials.gov Identifier: NCT01689246) [172] (Table 1). Begun in 2013, this trial is being conducted at more than 80 sites in North America, Australia, Europe, and Asia, using clinical (ADCS-CGIC), cognitive (ADAS-Cog 11), and safety measures as primary outcomes. The third Phase III trial is evaluating TRx0237 (200 mg/day) in 220 patients affected by the behavioral variant of frontotemporal dementia (bvFTD) and a MMSE above 20 (ClinicalTrials.gov Identifier: NCT01626378) [173] (Table 1). This trial adopted a modified version of the ADCS-CGIC scale as measure of clinical efficacy and the revised Addenbrooke's Cognitive Examination as cognitive measure. This trial was started in August 2013 and will involve 45 sites in North America, Europe, Australia, and Singapore. Finally, an open label extension study targets providing subjects who have completed participation in a Phase II or Phase III trials with TRx0237 continued access to therapy and to evaluate the long-term safety of the compound with an estimated study completion date of January 2017 (ClinicalTrials.gov Identifier: NCT02245568) [174] (Table 1). All three Phase III trials use “active placebo” tablets that include 4 mg of TRx0237 as a urinary and fecal colorant to help maintain blinding; therefore, the placebo group will receive a total of 8 mg/day of TRx0237. These Phase III trials are now fully recruited and results from these ongoing studies involving 250 centers in 22 countries around the world and 1,753 patients with mild to moderate AD or bvFTD are expected in early 2016.

## 8. Conclusion

In the last two decades, drug discovery and development efforts for AD research have been dominated by the “amyloid cascade hypothesis,” focusing on targets defined by this hypothesis and proposing amyloid as the main cause of neural death and dementia. Decreasing the formation or removing A*β* from the brain should attenuate dementia symptoms. Unfortunately, several clinical trials with anti-A*β* agents failed, challenging the hypothesis that A*β* accumulation is the initiating event in the pathological AD cascade and underscoring the need for novel therapeutic approaches and targets. In recent years, tau-based treatments for AD have become a point of increasing focus and current and previous investigational therapies can be grouped into four categories including tau-centric active and passive immunotherapies, microtubule-stabilizing agents, tau protein kinase inhibitors, and TAIs. Among these different approaches, small molecular weight compounds developed to inhibit formation of tau oligomers and fibrils by blocking tau-tau aggregation have already reached the clinic. Among TAIs, MT belongs to a class of diaminophenothiazines that have TAI activity* in vitro* [97, 131]. MTC, in which MT is dosed as the oxidized form MT^+^, was investigated in an exploratory Phase II dose-ranging double-blind clinical trial in 321 patients with mild to moderate AD [167]. The minimum effective dose was identified as 138 mg MT/day at both clinical and molecular imaging endpoints at 24 weeks. Treatment at this dose was found to prevent the decline in regional cerebral blood flow, particularly in medial temporal lobe structures and temporoparietal regions.

Given that the delivery of the highest dose of MT was impaired due to dose-dependent dissolution and absorption limitations, four Phase I studies [131] and two preclinical* in vitro *[132] and* in vivo* studies [133] were required to get to the bottom of the bioavailability limitations of the form of MT tested in the Phase II trial [167], setting out the basis for proceeding into Phase III trials with TRx0237 for AD treatment. Therefore, clinical development of MT for AD continues, along with a new form that is more bioavailable and less toxic at higher doses, called TRx0237, representing a new chemical entity that is distinct from both MTC and LMT. A broad-based approach to tau therapy appears favourable due to the numerous pathologic mechanisms for tau pathology. The potential contribution of tau conformation to inhibitory potency of TAIs suggests a route toward selectivity and an important target for future structural studies. In fact, identification of descriptors of inhibitory potency may provide a rational approach to compound optimization [95]. Therefore, the therapeutic benefit that has been reported for MT in Phase II stage and data from current Phase III trials will allow us to glean on the larger scale impact of TRx0237 and its therapeutic potential. However, the role of tau protein in AD pathogenesis should be better understood with future research including investigation of the mechanisms/pathways regulating the degradation of tau as determined by its posttranslational state, studies on soluble, nonaggregated forms of tau as a primary AD agent, exploring the role of tau as an enhancer of A*β*-induced degeneration, and clarifying the mechanisms by which pathological forms of tau may negatively impact mitochondrial biology.

In this direction, the observation that acetylation of soluble tau has important effects on the properties of tau, including its stability and aggregation, and that tau acetylation is elevated in patients at early and moderate Braak stages of tauopathy [23] has opened new possibilities of tau-based pharmacological approaches. A recent study has proved that tau acetylated at lysine 174 is one of the toxic species [175]. Increases in levels of this species have been associated with toxicity and cognitive impairment in transgenic mice. Conversely, blocking this acetylation with salsalate, a nonsteroidal anti-inflammatory drug, preserved cognition and led to improvements in pathology. Two to three months of treatment preserved hippocampal volume and reduced the number of NFTs by up to two-thirds. Moreover, treated animals maintained their memories better than their untreated littermates [175]. Because salsalate is an approved drug with a relatively good safety profile, it might be worth testing in AD patients.

## Figures and Tables

**Table 1:** Ongoing phase I–III randomized controlled trials (RCTs) of tau-directed drugs in clinical development for the treatment of Alzheimer's disease (AD).

Compound (company) Clinicaltrials.gov identifier	Mechanism of action	Estimated enrollment	Characteristics	Status
TRx0237 (LMTX) (TauRx Therapeutics Ltd.) NCT01626391	Tau aggregation inhibitor	9 patients already taking medications for probable mild to moderate AD (2012-2013)	TRx0237 tablets 250 mg/day (given as 125 mg bid) for 4 weeks	Phase II trial (completed)

TRx0237 (LMTX) (TauRx Therapeutics Ltd.) NCT01689233	Tau aggregation inhibitor	700 patients with probable mild AD (2012–2015)	TRx0237 100 mg tablets administered twice daily	Phase III trial (active not recruiting)

TRx0237 (LMTX) (TauRx Therapeutics Ltd.) NCT01689246	Tau aggregation inhibitor	833 patients with probable mild to moderate AD (2013–2016)	TRx0237 125 mg tablets administered twice daily	Phase III trial (active not recruiting)

TRx0237 (LMTX) (TauRx Therapeutics Ltd.) NCT01626378	Tau aggregation inhibitor	220 patients with behavioral variant of FTD (2013–2016)	TRx0237 100 mg tablet administered twice daily	Phase II trial (active not recruiting)

TRx0237 (LMTX) (TauRx Therapeutics Ltd.) NCT02245568	Tau aggregation inhibitor	Subjects who have completed participation in a Phase II or Phase III trial with TRx0237 continued access to therapy to evaluate the long-term safety of TRx0237 (2014–2017)	All subjects will initially be given 200 mg/day of TRx0237 administered twice daily. Thereafter, dosing is flexible (100 mg/day to 300 mg/day)	Open label Phase II trial (currently recruiting)

TPI-287 (University of California, San Francisco) NCT01966666	Microtubule-stabilizing agent	33 patients with mild to moderate AD (2013–2015)	The purpose of the study is to determine the highest dose of TPI-287 that is safe and tolerable when administered as an intravenous infusion	Phase I trial (currently recruiting)

AADvac1 (Axon Neuroscience SE) NCT01850238	Active tau-based immunotherapy	30 patients with mild to moderate AD (2013–2015)	Patients will receive 1 dose of AADvac1 per month over 3 months, for a total of 3 administrations	Phase I trial (completed)

AADvac1 (Axon Neuroscience SE) NCT02031198 FUNDAMANT	Active tau-based immunotherapy	This follow-up study continues to observe patients who have completed the Phase I trial of AADvac1, for another 18 months (2014–2017)	Patients who have received 6 doses in the previous trial will be given 1-2 booster doses of AADvac1 (2 if their antibody titers decline below those achieved in the previous trial). Patients who have received 3 doses in the previous trial will be given another 3 doses and then vaccinated with booster doses as above	18-month follow-up Phase I trial (active, not recruiting)

AADvac1 (Axon Neuroscience SE) NCT02579252 ADAMANT	Active tau-based immunotherapy	185 patients with mild AD (2016–2019)	Patients will receive 6 doses of AADvac1 in 4-week intervals and then 2 individual booster doses in 6-month intervals, for a total of 8 doses	Phase II trial (currently recruiting)

ACI-35 (AC Immune AG)	Active tau-based immunotherapy	Patients with mild to moderate AD (2013–2014)	This Phase I trial compared two doses of ACI-35 to investigate its safety, tolerability, and immunogenicity	Phase I trial (active, not recruiting)

RG7345 (RO6926496, MAb86) (Hoffmann-La Roche) NCT02281786	Passive tau-based immunotherapy	48 healthy subjects (January 2015–October 2015)	Single, ascending dose, intravenous administration	Phase I trial (active, not recruiting)

## References

[1] Alzheimer's Association (2015). 2015 Alzheimer's disease facts and figures. *Alzheimers & Dementia*.

[2] Schneider L. S., Mangialasche F., Andreasen N. (2014). Clinical trials and late-stage drug development for Alzheimer's disease: an appraisal from 1984 to 2014. *Journal of Internal Medicine*.

[3] Frisardi V., Solfrizzi V., Imbimbo B. P. (2010). Towards disease-modifying treatment of Alzheimer's disease: drugs targeting *β*-amyloid. *Current Alzheimer Research*.

[4] Panza F., Solfrizzi V., Imbimbo B. P., Logroscino G. (2014). Amyloid-directed monoclonal antibodies for the treatment of Alzheimer's disease: the point of no return?. *Expert Opinion on Biological Therapy*.

[5] Wischik C. M., Harrington C. R., Storey J. M. D. (2014). Tau-aggregation inhibitor therapy for Alzheimer's disease. *Biochemical Pharmacology*.

[6] Panza F., Solfrizzi V., Imbimbo B. P., Tortelli R., Santamato A., Logroscino G. (2014). Amyloid-based immunotherapy for Alzheimer's disease in the time of prevention trials: the way forward. *Expert Review of Clinical Immunology*.

[7] Panza F., Frisardi V., Solfrizzi V. (2012). Immunotherapy for Alzheimer's disease: from anti-*β*-amyloid to tau-based immunization strategies. *Immunotherapy*.

[8] Takashima A. (2010). Tau aggregation is a therapeutic target for Alzheimer's disease. *Current Alzheimer Research*.

[9] Buée L., Bussière T., Buée-Scherrer V., Delacourte A., Hof P. R. (2000). Tau protein isoforms, phosphorylation and role in neurodegenerative disorders. *Brain Research Reviews*.

[10] Lace G. L., Wharton S. B., Ince P. G. (2007). A brief history of *τ*: the evolving view of the microtubule-associated protein *τ* in neurodegenerative diseases. *Clinical Neuropathology*.

[11] Wischik C. M., Novak M., Thogersen H. C. (1988). Isolation of a fragment of tau derived from the core of the paired helical filament of Alzheimer disease. *Proceedings of the National Academy of Sciences of the United States of America*.

[12] Iqbal K., Liu F., Gong C.-X., Grundke-Iqbal I. (2010). Tau in Alzheimer disease and related tauopathies. *Current Alzheimer Research*.

[13] Bancher C., Brunner C., Lassmann H. (1989). Accumulation of abnormally phosphorylated *τ* precedes the formation of neurofibrillary tangles in Alzheimer's disease. *Brain Research*.

[14] Braak H., Thal D. R., Ghebremedhin E., Del Tredici K. (2011). Stages of the pathologic process in Alzheimer disease: age categories from 1 to 100 years. *Journal of Neuropathology & Experimental Neurology*.

[15] Braak H., Braak E. (1991). Neuropathological stageing of Alzheimer-related changes. *Acta Neuropathologica*.

[16] Braak H., Braak E. (1996). Evolution of the neuropathology of Alzheimer's disease. *Acta Neurologica Scandinavica, Supplement*.

[17] Braak H., Braak E. (1997). Frequency of stages of Alzheimer-related lesions in different age categories. *Neurobiology of Aging*.

[18] Mukaetova-Ladinska E. B., Garcia-Siera F., Hurt J. (2000). Staging of cytoskeletal and *β*-amyloid changes in human isocortex reveals biphasic synaptic protein response during progression of Alzheimer's disease. *The American Journal of Pathology*.

[19] Liu F., Iqbal K., Grundke-Iqbal I., Hart G. W., Gong C.-X. (2004). O-GlcNAcylation regulates phosphorylation of tau: a mechanism involved in Alzheimer's disease. *Proceedings of the National Academy of Sciences of the United States of America*.

[20] Goedert M., Spillantini M. G., Cairns N. J., Crowther R. A. (1992). Tau proteins of Alzheimer paired helical filaments: abnormal phosphorylation of all six brain isoforms. *Neuron*.

[21] Novak M., Kabat J., Wischik C. M. (1993). Molecular characterization of the minimal protease resistant tau unit of the Alzheimer's disease paired helical filament. *The EMBO Journal*.

[22] Guillozet-Bongaarts A. L., Garcia-Sierra F., Reynolds M. R. (2005). Tau truncation during neurofibrillary tangle evolution in Alzheimer's disease. *Neurobiology of Aging*.

[23] Min S.-W., Cho S.-H., Zhou Y. (2010). Acetylation of tau inhibits its degradation and contributes to tauopathy. *Neuron*.

[24] Kolarova M., García-Sierra F., Bartos A., Ricny J., Ripova D. (2012). Structure and pathology of tau protein in Alzheimer disease. *International Journal of Alzheimer's Disease*.

[25] Flores-Rodríguez P., Ontiveros-Torres M. A., Cárdenas-Aguayo M. C. (2015). The relationship between truncation and phosphorylation at the C-terminus of tau protein in the paired helical filaments of Alzheimer's disease. *Frontiers in Neuroscience*.

[26] Zhang Q., Zhang X., Sun A. (2009). Truncated tau at D421 is associated with neurodegeneration and tangle formation in the brain of Alzheimer transgenic models. *Acta Neuropathologica*.

[27] Saito M., Chakraborty G., Mao R.-F., Paik S.-M., Vadasz C., Saito M. (2010). Tau phosphorylation and cleavage in ethanol-induced neurodegeneration in the developing mouse brain. *Neurochemical Research*.

[28] Cohen T. J., Guo J. L., Hurtado D. E. (2011). The acetylation of tau inhibits its function and promotes pathological tau aggregation. *Nature Communications*.

[29] Guillozet-Bongaarts A. L., Cahill M. E., Cryns V. L., Reynolds M. R., Berry R. W., Binder L. I. (2006). Pseudophosphorylation of tau at serine 422 inhibits caspase cleavage: *in vitro* evidence and implications for tangle formation *in vivo*. *Journal of Neurochemistry*.

[30] Rohn T. T., Rissman R. A., Davis M. C., Kim Y. E., Cotman C. W., Head E. (2002). Caspase-9 activation and caspase cleavage of tau in the Alzheimer's disease brain. *Neurobiology of Disease*.

[31] Stadelmann C., Deckwerth T. L., Srinivasan A. (1999). Activation of caspase-3 in single neurons and autophagic granules of granulovacuolar degeneration in Alzheimer's disease: evidence for apoptotic cell death. *The American Journal of Pathology*.

[32] Sahara N., Maeda S., Murayama M. (2007). Assembly of two distinct dimers and higher-order oligomers from full-length tau. *European Journal of Neuroscience*.

[33] Kimura T., Fukuda T., Sahara N. (2010). Aggregation of detergent-insoluble tau is involved in neuronal loss but not in synaptic loss. *The Journal of Biological Chemistry*.

[34] Arrasate M., Mitra S., Schweitzer E. S., Segal M. R., Finkbeiner S. (2004). Inclusion body formation reduces levels of mutant huntingtin and the risk of neuronal death. *Nature*.

[35] Rubinsztein D. C. (2006). The roles of intracellular protein-degradation pathways in neurodegeneration. *Nature*.

[36] Díaz-Villanueva J. F., Díaz-Molina R., García-González V. (2015). Protein folding and mechanisms of proteostasis. *International Journal of Molecular Sciences*.

[37] Pritchard S. M., Dolan P. J., Vitkus A., Johnson G. V. W. (2011). The toxicity of tau in Alzheimer disease: turnover, targets and potential therapeutics. *Journal of Cellular and Molecular Medicine*.

[38] Frost B., Jacks R. L., Diamond M. I. (2009). Propagation of tau misfolding from the outside to the inside of a cell. *The Journal of Biological Chemistry*.

[39] Clavaguera F., Lavenir I., Falcon B., Frank S., Goedert M., Tolnay M. (2013). Prion-like templated misfolding in tauopathies. *Brain Pathology*.

[40] Clavaguera F., Bolmont T., Crowther R. A. (2009). Transmission and spreading of tauopathy in transgenic mouse brain. *Nature Cell Biology*.

[41] Asuni A. A., Boutajangout A., Quartermain D., Sigurdsson E. M. (2007). Immunotherapy targeting pathological tau conformers in a tangle mouse model reduces brain pathology with associated functional improvements. *The Journal of Neuroscience*.

[42] Masliah E., Rockenstein E., Adame A. (2005). Effects of *α*-synuclein immunization in a mouse model of Parkinson's disease. *Neuron*.

[43] Arriagada P. V., Growdon J. H., Hedley-Whyte E. T., Hyman B. T. (1992). Neurofibrillary tangles but not senile plaques parallel duration and severity of Alzheimer's disease. *Neurology*.

[44] Iqbal K., Gong C.-X., Liu F. (2014). Microtubule-associated protein tau as a therapeutic target in Alzheimer's disease. *Expert Opinion on Therapeutic Targets*.

[45] Pedersen J. T., Sigurdsson E. M. (2015). Tau immunotherapy for Alzheimer's disease. *Trends in Molecular Medicine*.

[46] Anand K., Sabbagh M. (2015). Early investigational drugs targeting tau protein for the treatment of Alzheimer's disease. *Expert Opinion on Investigational Drugs*.

[47] Khatoon S., Grundke-Iqbal I., Iqbal K. (1992). Brain levels of microtubule-associated protein *τ* are elevated in Alzheimer's disease: a radioimmuno-slot-blot assay for nanograms of the protein. *Journal of Neurochemistry*.

[48] Martin L., Latypova X., Wilson C. M. (2013). Tau protein kinases: involvement in Alzheimer's disease. *Ageing Research Reviews*.

[49] Gong C.-X., Iqbal K. (2008). Hyperphosphorylation of microtubule-associated protein tau: a promising therapeutic target for Alzheimer disease. *Current Medicinal Chemistry*.

[50] Plattner F., Angelo M., Giese K. P. (2006). The roles of cyclin-dependent kinase 5 and glycogen synthase kinase 3 in tau hyperphosphorylation. *The Journal of Biological Chemistry*.

[51] Wen Y., Planel E., Herman M. (2008). Interplay between cyclin-dependent kinase 5 and glycogen synthase kinase 3*β* mediated by neuregulin signaling leads to differential effects on tau phosphorylation and amyloid precursor protein processing. *Journal of Neuroscience*.

[52] Bhat R. V., Budd Haeberlein S. L., Avila J. (2004). Glycogen synthase kinase 3: a drug target for CNS therapies. *Journal of Neurochemistry*.

[53] Tolosa E., Litvan I., Höglinger G. U. (2014). A phase 2 trial of the GSK-3 inhibitor tideglusib in progressive supranuclear palsy. *Movement Disorders*.

[54] Del Ser T., Steinwachs K. C., Gertz H. J. (2013). Treatment of Alzheimer's disease with the GSK-3 inhibitor tideglusib: a pilot study. *Journal of Alzheimer's Disease*.

[55] Lovestone S., Boada M., Dubois B. (2015). ARGO investigators. A phase II trial of tideglusib in Alzheimer's disease. *Journal of Alzheimer's Disease*.

[56] Iqbal K., Liu F., Gong C.-X. (2014). Alzheimer disease therapeutics: focus on the disease and not just plaques and tangles. *Biochemical Pharmacology*.

[57] Kins S., Crameri A., Evans D. R. H., Hemmings B. A., Nitsch R. M., Götz J. (2001). Reduced protein phosphatase 2A activity induces hyperphosphorylation and altered compartmentalization of tau in transgenic mice. *The Journal of Biological Chemistry*.

[58] Arendt T., Holzer M., Fruth R., Brückner M. K., Gärtner U. (1995). Paired helical filament-like phosphorylation of tau, deposition of *β*/A4-amyloid and memory impairment in rat induced by chronic inhibition of phosphatase 1 and 2A. *Neuroscience*.

[59] Voronkov M., Braithwaite S. P., Stock J. B. (2011). Phosphoprotein phosphatase 2A: a novel druggable target for Alzheimer's disease. *Future Medicinal Chemistry*.

[60] Iqbal K., Alonso A. D. C., El-Akkad E. (2002). Significance and mechanism of Alzheimer neurofibrillary degeneration and therapeutic targets to inhibit this lesion. *Journal of Molecular Neuroscience*.

[61] Medina M., Avila J. (2015). Further understanding of tau phosphorylation: implications for therapy. *Expert Review of Neurotherapeutics*.

[62] Butler D., Bendiske J., Michaelis M. L., Karanian D. A., Bahr B. A. (2007). Microtubule-stabilizing agent prevents protein accumulation-induced loss of synaptic markers. *European Journal of Pharmacology*.

[63] Zhang B., Carroll J., Trojanowski J. Q. (2012). The microtubule-stabilizing agent, epothilone D, reduces axonal dysfunction, neurotoxicity, cognitive deficits, and alzheimer-like pathology in an interventional study with aged tau transgenic mice. *The Journal of Neuroscience*.

[64] https://globenewswire.com/news-release/2014/11/12/682514/10107850/en/Cortice-Biosciences-Announces-Results-From-Studies-Evaluating-Pipeline-Candidates-TPI-287-and-CRT-001-in-Preclinical-Models-of-Tauopathies-and-Alzheimer-s-Disease.html.

[65] University of California San Francisco (2000). A safety, tolerability, pharmacokinetics, pharmacodynamics and preliminary efficacy study of TPI-287 in Alzheimer's disease. *ClinicalTrials.gov*.

[66] University of California San Francisco Safety Study of TPI-287 to Treat CBS and PSP (TPI-287-4RT). *ClinicalTrials.gov*.

[67] Brunden K. R., Zhang B., Carroll J. (2010). Epothilone D improves microtubule density, axonal integrity, and cognition in a transgenic mouse model of tauopathy. *The Journal of Neuroscience*.

[68] Bristol-Myers Squibb (2000). *Study to Evaluate the Safety, Tolerability and the Effect of BMS-241027 on Cerebrospinal Fluid Biomarkers in Subjects with Mild Alzheimer's Disease*.

[69] Vulih-Shultzman I., Pinhasov A., Mandel S. (2007). Activity-dependent neuroprotective protein snippet NAP reduces tau hyperphosphorylation and enhances learning in a novel transgenic mouse model. *Journal of Pharmacology and Experimental Therapeutics*.

[70] Matsuoka Y., Jouroukhin Y., Gray A. J. (2008). A neuronal microtubule-interacting agent, NAPVSIPQ, reduces tau pathology and enhances cognitive function in a mouse model of Alzheimer's disease. *Journal of Pharmacology and Experimental Therapeutics*.

[71] Jouroukhin Y., Ostritsky R., Assaf Y., Pelled G., Giladi E., Gozes I. (2013). NAP (davunetide) modifies disease progression in a mouse model of severe neurodegeneration: protection against impairments in axonal transport. *Neurobiology of Disease*.

[72] Boxer A. L., Lang A. E., Grossman M. (2014). Davunetide in patients with progressive supranuclear palsy: a randomised, double-blind, placebo-controlled phase 2/3 trial. *The Lancet Neurology*.

[73] University of California. San Francisco (2000). *Davunetide (AL-108) in Predicted Tauopathies—Pilot Study*.

[74] (2000). Allon therapeutics. Study to evaluate safety, tolerability, and effect of AL208 on mild cognitive impairment following coronary artery bypass graft surgery. *ClinicalTrials.gov*.

[75] Rosenmann H., Grigoriadis N., Karussis D. (2006). Tauopathy-like abnormalities and neurologic deficits in mice immunized with neuronal tau protein. *Archives of Neurology*.

[76] Boutajangout A., Quartermain D., Sigurdsson E. M. (2010). Immunotherapy targeting pathological tau prevents cognitive decline in a new tangle mouse model. *The Journal of Neuroscience*.

[77] Boutajangout A., Wisniewski T. (2014). Tau-based therapeutic approaches for Alzheimer's disease—a mini-review. *Gerontology*.

[78] Kontsekova E., Zilka N., Kovacech B., Novak P., Novak M. (2014). First-in-man tau vaccine targeting structural determinants essential for pathological tau-tau interaction reduces tau oligomerisation and neurofibrillary degeneration in an Alzheimer's disease model. *Alzheimer's Research and Therapy*.

[79] Axon Neuroscience SE (2000). *Safety Study of AADvac1, A Tau Peptide-KLH-Conjugate Active Vaccine to Treat Alzheimer's Disease*.

[80] Taniguchi S., Suzuki N., Masuda M. (2005). Inhibition of heparin-induced tau filament formation by phenothiazines, polyphenols, and porphyrins. *The Journal of Biological Chemistry*.

[81] Axon Neuroscience SE (2000). *18-Months Safety Follow-Up Study of AADvac1, an Active Tau Vaccine for Alzheimer's Disease (FUNDAMANT)*.

[82] Axon Neuroscience SE (2000). *24 Months Safety and Efficacy Study of AADvac1 in Patients with Mild Alzheimer's Disease (ADAMANT)*.

[83] Theunis C., Crespo-Biel N., Gafner V. (2013). Efficacy and safety of a liposome-based vaccine against protein tau, assessed in tau.P301L mice that model tauopathy. *PLoS ONE*.

[84] Kayed R. (2010). Anti-tau oligomers passive vaccination for the treatment of Alzheimer disease. *Human Vaccines*.

[85] Kayed R., Head E., Thompson J. L. (2003). Common structure of soluble amyloid oligomers implies common mechanism of pathogenesis. *Science*.

[86] Boutajangout A., Ingadottir J., Davies P., Sigurdsson E. M. (2011). Passive immunization targeting pathological phospho-tau protein in a mouse model reduces functional decline and clears tau aggregates from the brain. *Journal of Neurochemistry*.

[87] Congdon E. E., Gu J., Sait H. B. R., Sigurdsson E. M. (2013). Antibody uptake into neurons occurs primarily via clathrin-dependent Fc*α* receptor endocytosis and is a prerequisite for acute tau protein clearance. *The Journal of Biological Chemistry*.

[88] Gu J., Congdon E. E., Sigurdsson E. M. (2013). Two novel Tau antibodies targeting the 396/404 region are primarily taken up by neurons and reduce Tau protein pathology. *The Journal of Biological Chemistry*.

[89] Buée L., Bussière T., Buée-Scherrer V., Delacourte A., Hof P. R. (2000). Tau protein isoforms, phosphorylation and role in neurodegenerative disorders. *Brain Research Reviews*.

[90] Collin L., Bohrmann B., Göpfert U., Oroszlan-Szovik K., Ozmen L., Grüninger F. (2014). Neuronal uptake of tau/pS422 antibody and reduced progression of tau pathology in a mouse model of Alzheimer's disease. *Brain*.

[91] Roche H.-L. A study of RO6926496 in healthy volunteers. *ClinicalTrials.gov*.

[92] Squibb B.-M. Multiple ascending dose study of intravenously administered BMS-986168 in patients with progressive supranuclear palsy (CN002-003). *ClinicalTrials.gov*.

[93] Bright J., Hussain S., Dang V. (2015). Human secreted tau increases amyloid-beta production. *Neurobiology of Aging*.

[94] Bristol-Myers Squibb (2000). *A Randomized, Double-Blind, Placebo-Controlled, Single Ascending Dose Study of Intravenously Administered BMS-986168 in Healthy Subjects*.

[95] Cisek K., Cooper G. L., Huseby C. J., Kuret J. (2014). Structure and mechanism of action of tau aggregation inhibitors. *Current Alzheimer Research*.

[96] Hampel H., Schneider L. S., Giacobini E. (2015). Advances in the therapy of Alzheimer's disease: targeting amyloid beta and tau and perspectives for the future. *Expert Review of Neurotherapeutics*.

[97] Wischik C. M., Edwards P. C., Lai R. Y. K., Roth M., Harrington C. R. (1996). Selective inhibition of Alzheimer disease-like tau aggregation by phenothiazines. *Proceedings of the National Academy of Sciences of the United States of America*.

[98] Necula M., Chirita C. N., Kuret J. (2005). Cyanine dye N744 inhibits tau fibrillization by blocking filament extension: implications for the treatment of tauopathic neurodegenerative diseases. *Biochemistry*.

[99] Chang E., Congdon E. E., Honson N. S., Duff K. E., Kuret J. (2009). Structure-activity relationship of cyanine tau aggregation inhibitors. *Journal of Medicinal Chemistry*.

[100] Pickhardt M., Biernat J., Khlistunova I. (2007). N-phenylamine derivatives as aggregation inhibitors in cell models of tauopathy. *Current Alzheimer Research*.

[101] Bulic B., Pickhardt M., Khlistunova I. (2007). Rhodanine-based tau aggregation inhibitors in cell models of tauopathy. *Angewandte Chemie—International Edition*.

[102] Pickhardt M., Larbig G., Khlistunova I. (2007). Phenylthiazolyl-hydrazide and its derivatives are potent inhibitors of *τ* aggregation and toxicity in vitro and in cells. *Biochemistry*.

[103] Pickhardt M., Gazova Z., Von Bergen M. (2005). Anthraquinones inhibit tau aggregation and dissolve Alzheimer's paired helical filaments in vitro and in cells. *The Journal of Biological Chemistry*.

[104] Crowe A., Huang W., Ballatore C. (2009). Identification of aminothienopyridazine inhibitors of tau assembly by quantitative high-throughput screening. *Biochemistry*.

[105] Li W., Sperry J. B., Crowe A., Trojanowski J. Q., Smith A. B., Lee V. M.-Y. (2009). Inhibition of tau fibrillization by oleocanthal via reaction with the amino groups of tau. *Journal of Neurochemistry*.

[106] Monti M. C., Margarucci L., Riccio R., Casapullo A. (2012). Modulation of tau protein fibrillization by oleocanthal. *Journal of Natural Products*.

[107] Casamenti F., Grossi C., Rigacci S., Pantano D., Luccarini I., Stefani M. (2015). Oleuropein aglycone: a possible drug against degenerative conditions. In vivo evidence of its effectiveness against Alzheimer's disease. *Journal of Alzheimer's Disease*.

[108] Wobst H. J., Sharma A., Diamond M. I., Wanker E. E., Bieschke J. (2015). The green tea polyphenol (−)-epigallocatechin gallate prevents the aggregation of tau protein into toxic oligomers at substoichiometric ratios. *FEBS Letters*.

[109] Crowe A., James M. J., Virginia M.-Y. Lee (2013). Aminothienopyridazines and methylene blue affect Tau fibrillization via cysteine oxidation. *The Journal of Biological Chemistry*.

[110] Morris G., Anderson G., Dean O. (2014). The glutathione system: a new drug target in neuroimmune disorders. *Molecular Neurobiology*.

[111] Schafer K. N., Cisek K., Huseby C. J., Chang E., Kuret J. (2013). Structural determinants of tau aggregation inhibitor potency. *The Journal of Biological Chemistry*.

[112] Kees F. (2013). Dimethyl fumarate: a janus-faced substance?. *Expert Opinion on Pharmacotherapy*.

[113] Bulic B., Pickhardt M., Mandelkow E. (2013). Progress and developments in tau aggregation inhibitors for Alzheimer disease. *Journal of Medicinal Chemistry*.

[114] Ahmad B., Lapidus L. J. (2012). Curcumin prevents aggregation in *α*-synuclein by increasing reconfiguration rate. *The Journal of Biological Chemistry*.

[115] Dolai S., Shi W., Corbo C. (2011). ‘Clicked’ sugar-curcumin conjugate: modulator of amyloid-*β* and tau peptide aggregation at ultralow concentrations. *American Chemical Society Chemical Neuroscience*.

[116] Landau M., Sawaya M. R., Faull K. F. (2011). Towards a pharmacophore for amyloid. *PLoS Biology*.

[117] Akoury E., Gajda M., Pickhardt M. (2013). Inhibition of tau filament formation by conformational modulation. *Journal of the American Chemical Society*.

[118] Masuda M., Suzuki N., Taniguchi S. (2006). Small molecule inhibitors of *α*-synuclein filament assembly. *Biochemistry*.

[119] Sengupta U., Guerrero-Muñoz M. J., Castillo-Carranza D. L. (2015). Pathological Interface between oligomeric alpha-synuclein and tau in synucleinopathies. *Biological Psychiatry*.

[120] Crowe A., Ballatore C., Hyde E., Trojanowski J. Q., Lee V. M.-Y. (2007). High throughput screening for small molecule inhibitors of heparin-induced tau fibril formation. *Biochemical and Biophysical Research Communications*.

[121] Bulic B., Pickhardt M., Mandelkow E.-M., Mandelkow E. (2010). Tau protein and tau aggregation inhibitors. *Neuropharmacology*.

[122] Dähne S. (1978). Color and constitution: one hundred years of research. *Science*.

[123] Schirmer R. H., Adler H., Pickhardt M., Mandelkow E. (2011). ‘Lest we forget you—methylene blue...’. *Neurobiology of Aging*.

[124] Wainwright M., Amaral L. (2005). The phenothiazinium chromophore and the evolution of antimalarial drugs. *Tropical Medicine and International Health*.

[125] Gaudette N. F., Lodge J. W. (2005). Determination of methylene blue and leucomethylene blue in male and female Fischer 344 rat urine and B6C3F1 mouse urine. *Journal of Analytical Toxicology*.

[126] Coulibaly B., Zoungrana A., Mockenhaupt F. P. (2009). Strong gametocytocidal effect of methylene blue-based combination therapy against falciparum malaria: a randomised controlled trial. *PLoS ONE*.

[127] Harvey B. H., Duvenhage I., Viljoen F. (2010). Role of monoamine oxidase, nitric oxide synthase and regional brain monoamines in the antidepressant-like effects of methylene blue and selected structural analogues. *Biochemical Pharmacology*.

[128] Peter C., Hongwan D., Küpfer A., Lauterburg B. H. (2000). Pharmacokinetics and organ distribution of intravenous and oral methylene blue. *European Journal of Clinical Pharmacology*.

[129] Müller T. (1998). Methylene blue supravital staining: an evaluation of its applicability to the mammalian brain and pineal gland. *Histology and Histopathology*.

[130] Baddeley T. C., McCaffrey J., Storey J. M. D. (2015). Complex disposition of methylthioninium redox forms determines efficacy in tau aggregation inhibitor therapy for Alzheimer's disease. *Journal of Pharmacology and Experimental Therapeutics*.

[131] Harrington C. R., Storey J. M. D., Clunas S. (2015). Cellular models of aggregation-dependent template-directed proteolysis to characterize tau aggregation inhibitors for treatment of Alzheimer disease. *The Journal of Biological Chemistry*.

[132] Melis V., Magbagbeolu M., Rickard J. E. (2015). Effects of oxidized and reduced forms of methylthioninium in two transgenic mouse tauopathy models. *Behavioural Pharmacology*.

[133] Dickey C. A., Ash P., Klosak N. (2006). Pharmacologic reductions of total tau levels; implications for the role of microtubule dynamics in regulating tau expression. *Molecular Neurodegeneration*.

[134] Jinwal U. K., Miyata Y., Koren J. (2009). Chemical manipulation of Hsp70 ATPase activity regulates tau stability. *The Journal of Neuroscience*.

[135] O'Leary J. C., Li Q., Marinec P. (2010). Phenothiazine-mediated rescue of cognition in tau transgenic mice requires neuroprotection and reduced soluble tau burden. *Molecular Neurodegeneration*.

[136] Congdon E. E., Wu J. W., Myeku N. (2012). Methylthioninium chloride (methylene blue) induces autophagy and attenuates tauopathy in vitro and in vivo. *Autophagy*.

[137] Xie L., Li W., Winters A., Yuan F., Jin K., Yang S.-H. (2013). Methylene blue induces macroautophagy through 5' adenosine monophosphate-activated protein kinase pathway to protect neurons from serum deprivation. *Frontiers in Cellular Neuroscience*.

[138] Akoury E., Pickhardt M., Gajda M., Biernat J., Mandelkow E., Zweckstetter M. (2013). Mechanistic basis of phenothiazine-driven inhibition of Tau aggregation. *Angewandte Chemie—International Edition*.

[139] Pfaffendorf M., Bruning T. A., Batink H. D., Van Zwieten P. A. (1997). The interaction between methylene blue and the cholinergic system. *British Journal of Pharmacology*.

[140] Mayer B., Brunner F., Schmidt K. (1993). Inhibition of nitric oxide synthesis by methylene blue. *Biochemical Pharmacology*.

[141] Chies A. B., Custódio R. C., de Souza G., Corrêa F. M. A., Pereira O. C. M. (2003). Pharmacological evidence that methylene blue inhibits noradrenaline neuronal uptake in the rat vas deferens. *Polish Journal of Pharmacology*.

[142] Vutskits L., Briner A., Klauser P. (2008). Adverse effects of methylene blue on the central nervous system. *Anesthesiology*.

[143] Ramsay R. R., Dunford C., Gillman P. K. (2007). Methylene blue and serotonin toxicity: inhibition of monoamine oxidase A (MAO A) confirms a theoretical prediction. *British Journal of Pharmacology*.

[144] Necula M., Breydo L., Milton S. (2007). Methylene blue inhibits amyloid A*β* oligomerization by promoting fibrillization. *Biochemistry*.

[145] Medina D. X., Caccamo A., Oddo S. (2011). Methylene blue reduces A*β* levels and rescues early cognitive deficit by increasing proteasome activity. *Brain Pathology*.

[146] Deiana S., Harrington C. R., Wischik C. M., Riedel G. (2009). Methylthioninium chloride reverses cognitive deficits induced by scopolamine: comparison with rivastigmine. *Psychopharmacology*.

[147] Riha P. D., Rojas J. C., Gonzalez-Lima F. (2011). Beneficial network effects of methylene blue in an amnestic model. *NeuroImage*.

[148] Paban V., Manrique C., Filali M., Maunoir-Regimbal S., Fauvelle F., Alescio-Lautier B. (2014). Therapeutic and preventive effects of methylene blue on Alzheimer's disease pathology in a transgenic mouse model. *Neuropharmacology*.

[149] Mori T., Koyama N., Segawa T. (2014). Methylene blue modulates *β*-secretase, reverses cerebral amyloidosis, and improves cognition in transgenic mice. *The Journal of Biological Chemistry*.

[150] Zakaria A., Hamdi N., Abdel-Kader R. M. (2016). Methylene blue improves brain mitochondrial ABAD functions and decreases A*β* in a neuroinflammatory Alzheimer’s disease mouse model. *Molecular Neurobiology*.

[151] Roy Choudhury G., Winters A., Rich R. M. (2015). Methylene blue protects astrocytes against glucose oxygen deprivation by improving cellular respiration. *PLoS ONE*.

[152] Wen Y., Li W., Poteet E. C. (2011). Alternative mitochondrial electron transfer as a novel strategy for neuroprotection. *The Journal of Biological Chemistry*.

[153] Poteet E., Winters A., Yan L.-J. (2012). Neuroprotective actions of methylene blue and its derivatives. *PLoS ONE*.

[154] Stack C., Jainuddin S., Elipenahli C. (2014). Methylene blue upregulates Nrf2/ARE genes and prevents tau-related neurotoxicity. *Human Molecular Genetics*.

[155] Hochgräfe K., Sydow A., Matenia D. (2015). Preventive methylene blue treatment preserves cognition in mice expressing full-length pro-aggregant human Tau. *Acta Neuropathologica Communications*.

[156] Mohideen S. S., Yamasaki Y., Omata Y., Tsuda L., Yoshiike Y. (2015). Nontoxic singlet oxygen generator as a therapeutic candidate for treating tauopathies. *Scientific Reports*.

[157] Al Mansouri A. S., Lorke D. E., Nurulain S. M. (2012). Methylene blue inhibits the function of *α*7-nicotinic acetylcholine receptors. *Central Nervous System & Neurological Disorders Drug Targets*.

[158] Visarius T. M., Stucki J. W., Lauterburg B. H. (1997). Stimulation of respiration by methylene blue in rat liver mitochondria. *FEBS Letters*.

[159] van Bebber F., Paquet D., Hruscha A., Schmid B., Haass C. (2010). Methylene blue fails to inhibit Tau and polyglutamine protein dependent toxicity in zebrafish. *Neurobiology of Disease*.

[160] Hosokawa M., Arai T., Masuda-Suzukake M. (2012). Methylene Blue reduced abnormal tau accumulation in P301L tau transgenic mice. *PLoS ONE*.

[161] Spires-Jones T. L., Friedman T., Pitstick R. (2014). Methylene blue does not reverse existing neurofibrillary tangle pathology in the rTg4510 mouse model of tauopathy. *Neuroscience Letters*.

[162] Cavaliere P., Torrent J., Prigent S. (2013). Binding of methylene blue to a surface cleft inhibits the oligomerization and fibrillization of prion protein. *Biochimica et Biophysica Acta*.

[163] Yamashita M., Nonaka T., Arai T. (2009). Methylene blue and dimebon inhibit aggregation of TDP-43 in cellular models. *FEBS Letters*.

[164] Arai T., Hasegawa M., Nonoka T. (2010). Phosphorylated and cleaved TDP-43 in ALS, FTLD and other neurodegenerative disorders and in cellular models of TDP-43 proteinopathy. *Neuropathology*.

[165] MacKenzie I. R. A., Neumann M., Cairns N. J., Munoz D. G., Isaacs A. M. (2011). Novel types of frontotemporal lobar degeneration: beyond Tau and TDP-43. *Journal of Molecular Neuroscience*.

[166] Wischik C. M., Staff R. T., Wischik D. J. (2015). Tau aggregation inhibitor therapy: an exploratory phase 2 study in mild or moderate Alzheimer's disease. *Journal of Alzheimer's Disease*.

[167] TauRx Therapeutics Ltd Open label study of TRx0014 in Alzheimer's disease. *ClinicalTrials.gov*.

[168] Rager T., Geoffroy A., Hilfiker R., Storey J. M. D. (2012). The crystalline state of methylene blue: a zoo of hydrates. *Physical Chemistry Chemical Physics*.

[169] Melis V., Zabke C., Stamer K. (2015). Different pathways of molecular pathophysiology underlie cognitive and motor tauopathy phenotypes in transgenic models for Alzheimer's disease and frontotemporal lobar degeneration. *Cellular and Molecular Life Sciences*.

[170] TauRx Therapeutics (2000). *Safety Study of TRx0237 in Patients Already Taking Medications for Mild and Moderate Alzheimer's Disease*.

[171] TauRx Therapeutics Ltd (2000). *Safety and Efficacy Study Evaluating TRx0237 in Subjects with Mild Alzheimer's Disease*.

[172] TauRx Therapeutics (2000). *Safety and Efficacy Study Evaluating TRx0237 in Subjects with Mild to Moderate Alzheimer's Disease*.

[173] TauRx Therapeutics (2000). *Safety and Efficacy Study Evaluating TRx0237 in Subjects with Behavioral Variant Frontotemporal Dementia (bvFTD)*.

[174] TauRx Therapeutics (2000). *Open-Label Study of TRx0237 in Subjects With Alzheimer's Disease or Behavioral Variant Frontotemporal Dementia (bvFTD)*.

[175] Min S.-W., Chen X., Tracy T. E. (2015). Critical role of acetylation in tau-mediated neurodegeneration and cognitive deficits. *Nature Medicine*.

